# Antiglycating potential of *Zingiber officinalis* and delay of diabetic cataract in rats

**Published:** 2010-08-10

**Authors:** Megha Saraswat, Palla Suryanarayana, Paduru Yadagiri Reddy, Madhoosudan A. Patil, Nagalla Balakrishna, Geereddy Bhanuprakash Reddy

**Affiliations:** 1 Biochemistry Division, National Institute of Nutrition, Hyderabad, India; 2 Statistics Division, National Institute of Nutrition, Hyderabad, India

## Abstract

**Purpose:**

Advanced glycation end products (AGE) are associated in the development of several pathophysiologies including diabetic cataract. Earlier we have reported that some common dietary agents have antiglycating activity and ginger (*Zingiber officinalis*) was one of the few prominent agents that effectively prevented AGE formation in vitro. In this study we investigated the potential of ginger to prevent diabetic cataract in rats.

**Methods:**

Diabetes was induced in Wistar-NIN rats by intraperitoneal injection of streptozotocin (35 mg/kg bodyweight) and the control rats received vehicle alone. While a set of diabetic animals received AIN-93 diet, another set received either 0.5 or 3% ginger in their diet for a period of two months. Cataract progression was monitored by slit-lamp biomicroscope. At the end of two months, the animals were sacrificed to evaluate non-enzymatic glycation and osmotic stress in the eye lens.

**Results:**

Slit-lamp examination revealed that feeding of ginger not only delayed the onset but also the progression of cataract in rats. Molecular analyses indicated that feeding of ginger significantly inhibited the formation of various AGE products including carboxymethyl lysine in the eye lens. In addition, ginger also countered hyperglycemia-induced osmotic stress in the lens.

**Conclusions:**

The results indicated that ginger was effective against the development of diabetic cataract in rats mainly through its antiglycating potential and to a lesser extent by inhibition of the polyol pathway. Thus, ingredients of dietary sources, such as ginger, may be explored for the prevention or delay of diabetic complications.

## Introduction

The statistical facts indicate that the world, particularly South-East Asia including India, is facing a growing diabetes epidemic of potentially devastating proportions [1,2]. Prolonged exposure to chronic hyperglycemia, without proper management, can lead to various short-term and long-term secondary complications, which represent the main cause of morbidity and mortality in diabetic patients. Cataract, characterized by the opacification of the eye lens that interferes with transmission of light onto the retina, is one of the earliest secondary complications of diabetes. Cataract is the leading cause of visual impairment across the world and studies indicate that the incidence of cataract is much higher in diabetic than in non-diaetic individuals [3,4].

Considerable evidence suggests that the long-term pathological sequel of diabetes is a result of the accumulation of tissue macromolecules that have been progressively modified by non-enzymatic glycation [5-7]. Glycation is a non-enzymatic reaction between reducing sugar and free amino group of the protein (predominantly the ε-amino group of lysine and the guanidine group of arginine) forming an Amadori product [5-7]. The latter then undergoes a series of complex reactions (oxidative and nonoxidative) resulting in the formation of advanced glycation end products (AGE). Non-enzymatic glycation of lens protein has been considered to be one of the major factors responsible for diabetic cataract [7-10], which alters protein conformation and stability, induces protein aggregation and cross-linking, and leads to protein insolubilization [11-14]. Studies also show the formation of AGE not only plays a key role in the development of diabetic cataract but also in the several pathophysiologies associated with aging and diabetes such as arthritis, atherosclerosis, chronic renal insufficiency, Alzheimer disease, nephropathy, and neuropathy [5,15-17]. This raises the possibility that inhibition of AGE formation may prevent the progression of diabetic complications including diabetic cataract.

Aminoguanidine, a nucleophilic hydrazine compound, has been extensively studied for its AGE inhibition [5,18,19]. However, the trial was terminated due to various safety concerns [5,18]. In case of cataract, aminoguanidine could delay the progression of opacification of lens only in moderately diabetic rats [20]. A wide variety of agents such as pyridoxamine, carnosine, carnitine, lipoic acid, taurine, OPB-9195, and phenyl thiazolium bromide have been investigated in several in vitro and in vivo studies [5,21-23] and some of these agents have been tested against cataract in animal model. However, except pyridoxamine, none have progressed, as yet, to the stage of clinical trials. Thus, there is a need for developing new antiglycating agents combining higher levels of efficacy, selectivity and safety in humans.

In the context of a need for developing and testing new antiglycating agents, we have evaluated several traditional medicines and some common dietary agents and found that some spice principles have the potential to inhibit AGE formation under in vitro conditions [23] and in animal models [24]. Among them ginger was one of the agents that significantly prevented AGE formation in vitro [23]. Although, ginger *(Zingiber officinale)* is one of the commonly used spices for culinary purposes, it has been used to treat a variety of conditions such as arthritis, rheumatism, sprains, muscular aches, pains, sore throats, constipation, and indigestion in the traditional medicinal system of Asia [25]. Although, the hypoglycemic activity of ginger was found in some studies [26,27], variable results have been reported in different studies [28,29]. Since we found obvious antiglycating activity with aqueous extract of ginger in vitro [23], we investigated the significance of its antiglycating potential under in vivo conditions using streptozotocin (STZ)-induced diabetic cataract model in rats.

## Methods

### Materials

Streptozotocin (STZ), NADPH, NADH, 2-thiobarbituric acid (TBA), 1,1.3,3-tetraethoxy propane (TEP), DL-glyceraldehyde lithium sulfate, β-mercaptoethanol, glutathione, 2,4-dinitrophenylhydrazine (DNPH), EDTA, BSA, methylglyoxal (MGO), Freund’s complete and incomplete adjuvant, m-aminophenyl boronic acid, and HRP-conjugated goat anti-rabbit antibody were obtained from Sigma-Aldrich (St. Louis, MO). Glyoxylic acid and sodium cyanoborohydride were purchased from ICN (Orangeburg, NY). Immobilon-NC membrane was from Millipore (Bedford, MA) and protein A-sepharose beads were obtained from Amersham Biosciences (Piscataway, NJ). All other chemicals and solvents were of analytical grade and were obtained from local companies.

### Preparation of AGE antigens

AGE-BSA, MGO-BSA, and CML (carboxymethyl lysine)-BSA were prepared as described previously  [12,23,24]. Briefly, for AGE-BSA, BSA (50 mg/ml) was incubated with 1 M glucose in 0.2 M phosphate buffer (pH 7.4) containing 0.05 % sodium azide at 37 °C for 90 days. For MGO-BSA, BSA (50 mg/ml) was incubated with 0.5 M methylglyoxal in 100 mM sodium phosphate buffer (pH 7.5) at 37 °C in dark for 3 days. Bovine serum albumin (BSA, 50 mg/ml) was incubated with 0.045 M glyoxylic acid and 0.15 M sodium cyanoborohydride in 0.2 M sodium phosphate buffer (pH7.8) for 24 h at 37 °C for the preparation of CML-BSA. Low molecular weight reactants and unbound sugars were removed by extensive dialysis.

### Production of polyclonal anti-AGE antisera

Production of polyclonal antiserum against AGE-BSA, MGO-BSA, and CML-BSA was described previously  [23]. Briefly, 8–12 week old rabbits were immunized with respective AGE-protein antigens (1 mg/ml) in Freund's complete adjuvant and subsequently three boosters were given at 3-week intervals in Freund's incomplete adjuvant. The rabbits were bled after the last booster, and the serum was collected. Antiserum was partially purified by ammonium sulfate fractionation followed by DEAE-sepharose anion exchange chromatography to obtain IgG rich fraction [12,23,24].

### Preparation of ginger powder

Rhizomes of fresh wet ginger (*Zingiber officinalis*) were collected from a local market in one lot and freeze-dried using a lyophilizer (Virtis-Freeze Mobile; SP Scientific, Stone Ridge, NY). The freeze-dried material was powdered using a grinder-mixer and was mixed with AIN-93M diet in required doses and fed to the rats.

### Experimental design and dietary regimen

Two-month-old male WNIN (Wistar-NIN) rats with an average bodyweight of 220±17 g (obtained from the National Centre for Laboratory Animal Sciences, Hyderabad, India) were used in the study. All the animals were fed AIN-93 diet ad libitum. The control (Group I; n=9) rats received 0.1 M citrate buffer, pH 4.5 as a vehicle whereas the experimental rats received a single intraperitoneal injection of STZ (35 mg/kg) in citrate buffer. After 72 h, fasting blood glucose levels were monitored. Animals having blood glucose levels <150 mg/dl were excluded from the experiment and the rest were distributed into three groups (Groups II-IV). Animals in Group II (n=12) received only AIN-93 diet whereas Group III (n=9) and Group IV (n=9) animals received the AIN-93 diet containing 0.5% and 3% freeze-dried powder of wet ginger, respectively.

### Animal care

Institutional and national guidelines for the care and use of animals were followed and all experimental procedures involving animals were approved by the IAEC (institutional animal ethical committee) of the National Institute of Nutrition. Animals were housed in individual cages in a temperature (22 °C) and humidity-controlled room with a 12 h light/dark cycle. All the animals had free access to water. Food intake (daily) and bodyweights (weekly) were monitored. We adhered to the ARVO Statement for the Use of Animals in Ophthalmic and Vision Research.

### Slit lamp examination and cataract grading

Eyes were examined every week using a slit lamp biomicroscope (Kowa Portable; Kowa, Ltd., Tokyo, Japan) on dilated pupils. Initiation and progression of lens opacity was graded into five categories as described previously  [30]. Briefly, lens opacity was graded as clear - clear lenses with no vacuoles; stage 1 - vacuoles cover approximately one-half of the surface of the anterior pole; stage 2 - some vacuoles have disappeared and the cortex haziness; stage 3 - a hazy cortex remains and dense nuclear opacity is present; stage 4 - a mature cataract.

### Blood and lens collection and processing

Blood was collected once a week from the retro-orbital plexus for glucose and insulin estimation. At the end of 9 weeks, animals were sacrificed by CO_2_ asphyxiation, and lenses were dissected by the posterior approach  [31]. Briefly, a small incision was made on the posterior side of the eye with the scissors. The lenses were collected by pressing with tweezers against the side of the eye opposite of the incision and stored at −70 °C until further analysis. A 10% homogenate was prepared from 3 to 4 pooled lenses in 50 mM phosphate buffer (pH 7.4). Some of the biochemical estimations such as malondialdehyde (MDA), sorbitol cannot be done with two lenses of each rat as the rat lens weigh about 40 mg. Hence we pooled 3–4 lenses for making homogenate not only to estimate MDA and sorbitol but also for other biochemical estimations from the same pool. All the biochemical parameters were analyzed in the soluble fraction of the lens homogenate (15,000× g at 4 °C) except for lens malondialdehyde (MDA) and sorbitol which were determined in the total homogenate.

### Biochemical estimations

Serum glucose was measured by the glucose oxidase-peroxidase (GOD-POD) method using a commercially available kit (BioSystems S.A.Costa Brava 30, Barcelona, Spain) and serum insulin was measured by a RIA kit (BRITE-DAE, Mumbai, India). Lens protein carbonyl content and the activities of aldose reductase (ALR2) and sorbitol dehydrogenase (SDH) were determined according to methods described previously [23,30]. Total, soluble, and insoluble protein was assayed by the Lowry method, with BSA as a standard.

### SDS–PAGE of lens proteins

The subunit profile and cross-linking of lens soluble proteins were analyzed on 12% polyacrylamide gels in the presence of SDS under reducing conditions [24,30].

### Immunodetection of AGE-antigens in the soluble fraction of lens

Lens soluble proteins were resolved under reducing conditions on 12% SDS–PAGE, proteins were transferred onto a nitrocellulose membrane (NC) and NC was blocked with 5% skimmed milk powder. NC membrane was incubated with partially purified antisera of AGE-BSA, MGO-BSA and CML-BSA (1:100 dilution) later with HRP-conjugated goat anti-rabbit antibody (1:500). Subsequently, detection was performed with diaminobenzidine in the presence of hydrogen peroxide.

### Immunoprecipitation of CML and MGO-AGE antigens in the insoluble fraction of lens

Immunoprecipitation (IP) was performed using 1 mg of reconstituted insoluble fraction of lens in 500 μl of IP buffer (50 mM sodium phosphate buffer pH 7.4). Partially purified polyclonal antisera of CML-BSA or MGO-BSA was added at 1:100 dilution in IP buffer and incubated at 4 °C overnight. To this, 50 μl of slurry of Protein-A was added and incubated for 3 h at 4 °C. Immunocomplex was collected by centrifugation at 10,000× g for 1 min at 4 °C and complex was washed 3 times with 1 ml of IP buffer, spinning at 10,000× g each time for 1 min. Finally, pellet was washed with 50 mM Tris-HCl pH 7.9 and re-suspended in sample buffer. Immunoblotting and detection was performed as described above using anti-CML-BSA or anti-MGO-BSA antiserum (1:100 dilution).

### Estimation of glycated protein by boronate affinity chromatography

The percentage of glycated protein was estimated in the soluble fraction of lens as described previously  [23,24]. Briefly, 5 mg of glycated lens protein was passed through a phenyl boronate affinity column equilibrated with 0.25 M ammonium acetate buffer (pH 8.5) containing 0.05 M MgCl_2_. The unbound fraction containing non-glycated protein was washed with the above buffer, while bound glycated protein was eluted using 0.1 M 2-amino-2-hydroxymethyl-propane-1,3-diol-HCl (pH 7.5) containing 0.2 M sorbitol.

### Statistical analysis

Trajectories were estimated using mixed model analysis using SPSS software version 15.0 (SPSS Inc., Chicago, IL) to test the statistical significance of delay of cataract progression due to ginger feeding. One-way ANOVA was used for testing statistical significance between groups of data and individual pair difference was tested by means of Duncan’s multiple-range test. Heterogeneity of variance was tested by the nonparametric Mann–Whitney test where a p<0.05 was considered as significant.

## Results

### Food intake and bodyweights

As reported by us earlier [24,30,32], there was an increase in the food intake in all the diabetic animals compared with the control animals (data not shown). Studies suggest that a deficiency of hormones, particularly insulin and leptin, causes hyperphagia in streptozotocin (STZ) diabetic rats by altering the balance of hypothalamic neuropeptides [33-35], though the signaling mechanisms causing diabetic hyperphagia are still incompletely understood. Irrespective of food intake, the dynamics of amino nitrogen conversion is changed in a way that favors protein catabolism of diabetic animals causing weight loss [36]. Despite the increased food intake, the bodyweight of diabetic animals decreased significantly (mean±SEM bodyweight at the end of experiment, 208±3.28 g; p<0.001) when compared to control animals (290±2.96 g). However feeding of ginger to diabetic rats (Group III and IV) did not normalize the bodyweights to a significant extent (217±2.55 g; p<0.001 and 225±5.50 g; p<0.001, respectively).

### Cataract progression

**Figure 1:**
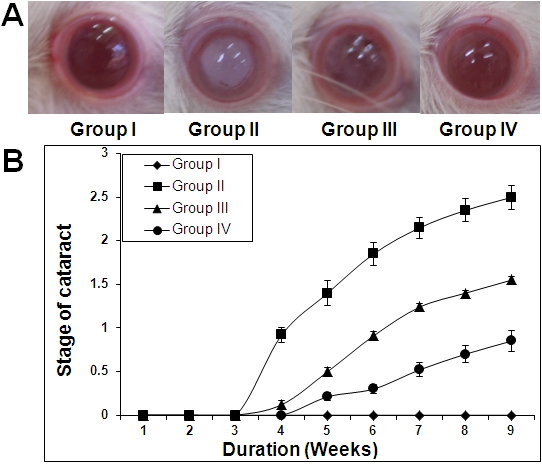
Delay of diabetic cataract in rats by ginger. **A**: Representative photographs of lens from each group at the end of 9 weeks. **B**: Quantitative representation of cataract progression in each group with time. Stage of cataract in each group was averaged at a given time and the average stage of cataract along with standard error was plotted as a function of time.

While the onset of cataract due to hyperglycemia was observed in diabetic animals after four weeks of STZ injection and progressed to mature cataract by eight weeks in untreated diabetic lenses, feeding of ginger delayed both onset and progression of cataract in diabetic rats in a dose dependent manner (Figure 1A). Since lenses were in different stages of cataract in a given group at a given time, the number lenses in each stage of cataract for different groups at the end of experiment was shown (Table 1). Further, we have averaged the stages at the given time at a given group, to see the onset and progression of cataract in an empirical manner in all the groups and presented with duration of diabetes (Figure 1B). Interestingly, there was a delay in the onset of cataract in ginger fed groups as compared to untreated diabetic group. At the end of the nine weeks, the severity of cataract was significantly lower in ginger-fed groups, particularly with 3% ginger, than in untreated diabetic rats. All the lenses in control group appeared to be normal and free of opacities during the experimental period. Based on the trajectories (0, 1.169, 0.620, and 0.241, respectively for Group I; Group II; Group III, and Group IV) which are highly significant (p<0.001) at all the time intervals, the data suggests that progression of lens abnormalities and maturation of cataract due to STZ-induced hyperglycemia were significantly delayed with feeding of ginger in a dose dependent manner (Figure 1B). The doses of ginger used in the study were based on a pilot study involving a small number of rats (data not shown).

**Table 1:** Effect of ginger on progression of diabetic cataract.

**Cataract stage**	**Group I**	**Group II**	**Group III**	**Group IV**
Clear	18	0	0	0
Stage <1	0	0	7	14
Stage 1–2	0	7	9	4
Stage 2–3	0	13	2	0
Stage 3–4	0	4	0	0

Number of lenses in each stage of cataract for different groups at the end of experiment.

### Blood glucose and insulin levels

**Figure 2:**
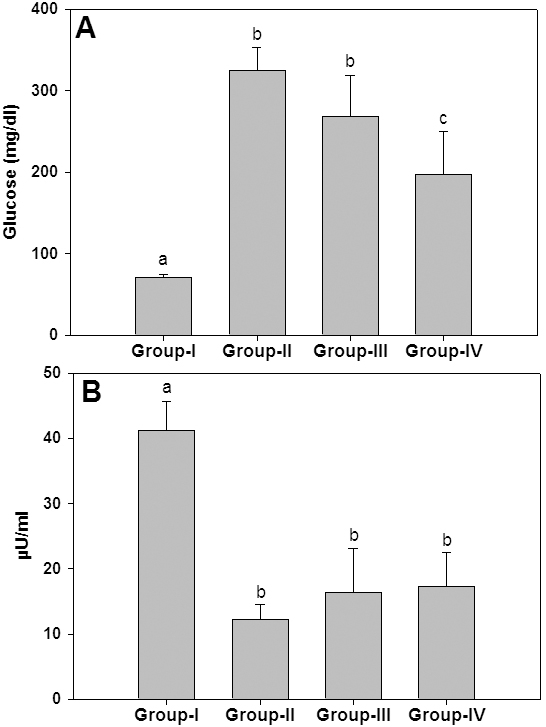
Blood glucose and insulin levels. Effect of ginger on blood glucose (**A**) and insulin levels (**B**) at the end of study was shown. Data are mean±SD of all the animals in a group. Different superscripts above the bars denote that data are significantly different among the groups.

We have assessed blood glucose and insulin levels to understand whether ginger ameliorated cataract onset and progression by reducing STZ-induced hyperglycemia. As expected blood glucose levels were elevated and insulin levels were lowered significantly in untreated diabetic animals compared to control rats (Figure 2). Though blood glucose levels were decreased to a marginal (low dose) to moderate (high dose) level due to ginger feeding to diabetic rats, insulin levels were unaffected by both levels of ginger (Figure 2). Thus, a modest lowering of blood glucose levels in diabetic rats by ginger feeding is supported by unaltered insulin levels. However, it should be noted that the plasma glucose levels in ginger-fed diabetic rats are considerably higher than plasma glucose levels (150 mg/dl) which are shown to induce cataract in WNIN rats after 4–5 weeks as seen in this study and earlier studies [24,30,32]. Therefore, delay of cataract progression in diabetic rats due to ginger feeding could be attributed to its potential to modulate pathways that are linked to the development of diabetic complications including cataract instead of mere partial lowering of blood glucose level.

### Molecular basis for the delay of cataract

To investigate the possible mechanisms by which ginger delayed the onset and progression of STZ-induced diabetic cataract, we have studied various biochemical mechanisms related to cataractogenesis. In view of in vitro antiglycating potential of ginger [23], we have focused on non-enzymatic glycation mediated effects. In addition we have also investigated the status of polyol pathway and oxidative stress as there is a cross-talk between these pathways [37,38].

### Protein glycation

**Figure 3:**
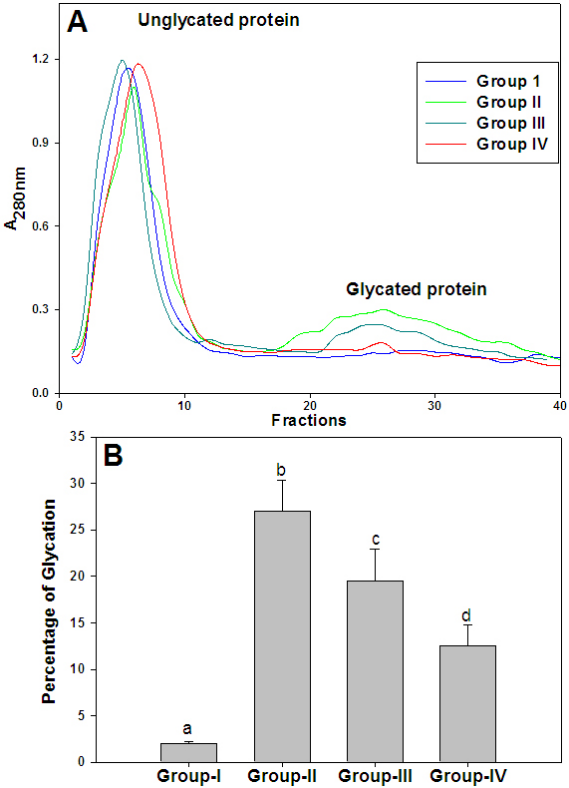
Glycated protein in soluble fraction of lens. **A**: Amount of glycated protein in soluble protein fraction of different groups was assayed by phenyl boronate affinity chromatography as described in the Methods section. **B**: Percent of glycated protein in soluble proteins of lens. Data are mean±SD (n=4). Different superscripts above the bars denote that data are significantly different among the groups.

**Figure 4:**
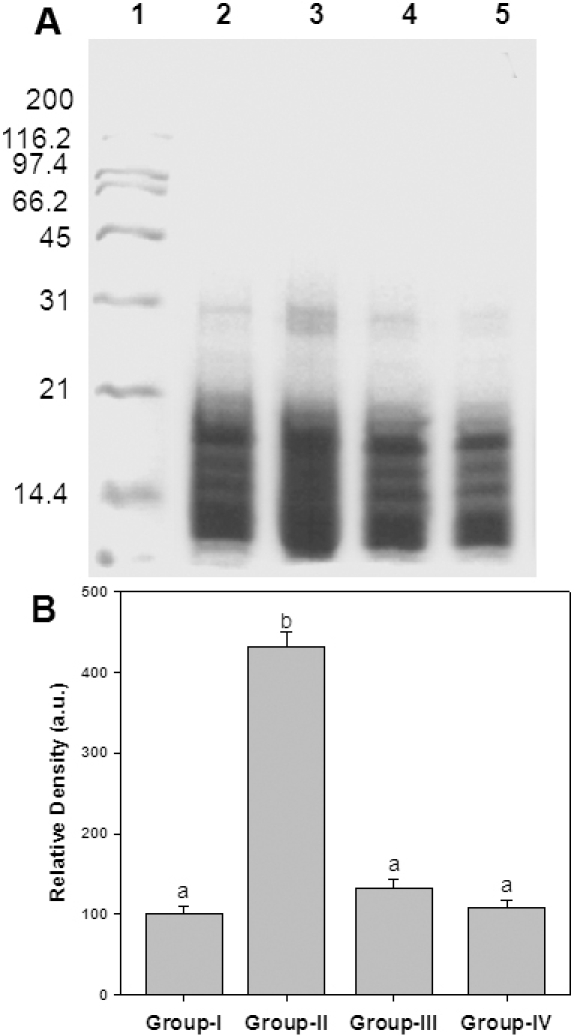
Sub unit profile and protein cross-linking of soluble fraction of lens. **A**: Soluble protein was loaded onto a 12% polyacrylamide gel under reducing conditions. Molecular weight standards in kDa are indicated alongside of the gel. Lane 1- Molecular weight markers; Lane 2 - Group I; Lane 3 - Group II; Lane 4 - Group III; Lane 5 - Group IV. **B**: Densitometric quantification of cross links. Data are mean±SD (n=4). Intensity of protein bands above 31 kDa was quantified considering the intensity of Group I as 100%. Different superscripts above the bars denote that data are significantly different among the groups.

To understand that ginger feeding to diabetic rats could reduce the glycation, the percentage of glycated protein in the lens was measured using boronate affinity chromatography (Figure 3). While the percentage of glycated protein in untreated diabetic rats was about 27%, ginger feeding resulted in a reduction of glycated protein in a dose depended manner (Figure 3). Further, these results corroborate with our previous findings of in vitro antiglycating potential of ginger. Despite their heterogeneity, a propensity to form covalent cross links is the common consequence of AGE, which leads to the formation of high molecular weight aggregates on proteins. Hence, we have monitored the cross-links of lens proteins and also high molecular weight (HMW) aggregates by SDS–PAGE. While the SDS–PAGE pattern of soluble protein showed an increased proportion of cross linked and aggregated proteins with the appearance of non-disulfide dimers of molecular mass 45 kDa in the diabetic rat lens soluble proteins compared with control, ginger fed groups showed a profile similar to control group (Figure 4). Alteration in the protein profile due to HMW aggregates lead to the insolubilization of protein that have been considered to be the ultimate change that results in lens opacification. Therefore, we have also analyzed total, soluble, and insoluble protein content in all the groups. There was a significant decrease in both total and soluble protein in the untreated diabetic group compared with the control group. Generally during progression and subsequent maturation of cataract, while there is a leakage of proteins from the lens due to osmotic stress in addition to degradation of lens proteins which is the main cause of decreased total protein content, there is an insolubilization of available soluble protein due to mechanisms mentioned above. Feeding the rats with ginger improved the total and soluble protein levels, which is in agreement with the delay of onset of cataract in those groups (Table 2).

**Table 2:** Effect of ginger on protein content of rat lens.

**Parameter**	**Group I**	**Group II**	**Group III**	**Group IV**
Total protein (mg/g lens)	480^a^±32.4	384^b^±22.7	434^c^±31.2	465^a^±35.8
Soluble protein (mg/g lens)	360^a^±27.3	198^b^±25.45	274^c^±40.5	310^c^±29.4
Soluble protein (%)	75.0	51.5	63.1	66.6

The total and soluble protein in the lens of different groups was estimated by the Lowry’s method and the percentage soluble protein content was derived from the estimated values. The data are mean±SD (n=4). Different superscripts denote that data are significantly different among the groups.

**Figure 5:**
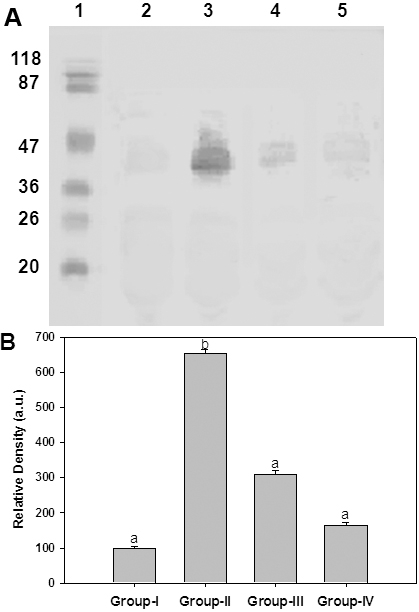
Immunodetection of glucose derived AGEs in the soluble portion of lens protein. **A**: Representative western blot profile of soluble lens protein probed with anti-AGE-BSA antibodies. Lane 1: Molecular weight markers, Lane 2: Group I, Lane 3: Group II, Lane 4: Group III, Lane 5: Group IV. **B**: Densitometry analysis of AGE-BSA. Intensity of AGE-BSA signals was quantified considering the intensity of lane 2 in upper panel as 100%. Data in lower panel are mean±SEM of three independent experiments and different superscripts denote that data are significantly different among the groups.

**Figure 6:**
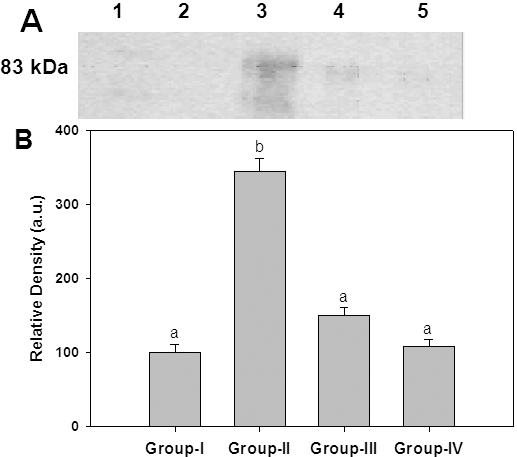
Immunodetection of CML in the insoluble portion of lens. **A**: Representative western blot profile of insoluble lens protein probed with anti-CML-BSA antibodies. Lane 1: Molecular weight markers, Lane 2: Group I, Lane 3: Group II, Lane 4: Group III, Lane 5: Group IV. **B**: Densitometry analysis of CML-BSA. Intensity of CML-BSA signals was quantified considering the intensity of lane 2 in upper panel as 100%. Data in lower panel are mean±SEM of three independent experiments and different superscripts denote that data are significantly different among the groups.

**Figure 7:**
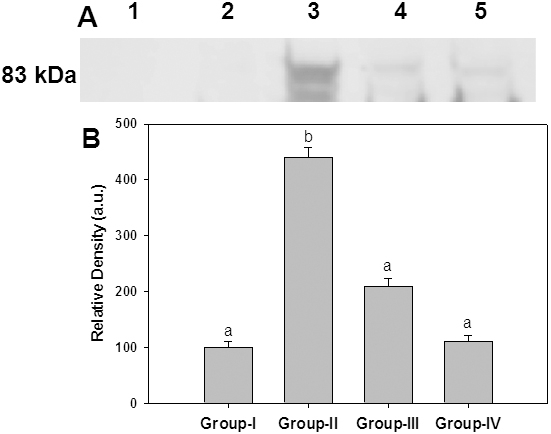
Immunodetection of MGO-AGE in the insoluble portion of lens. **A**: Representative western blot profile of insoluble lens protein probed with anti-MGO-BSA antibodies. Lane 1: Molecular weight markers, Lane 2: Group I, Lane 3: Group II, Lane 4: Group III, Lane 5: Group IV. **B**: Densitometry analysis of MGO-BSA. Intensity of MGO-BSA signals was quantified considering the intensity of lane 2 in upper panel as 100%. Data in lower panel are mean±SEM of three independent experiments and different superscripts denote that data are significantly different among the groups.

**Figure 8:**
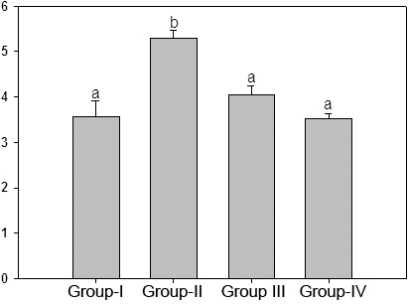
Protein carbonyl content in soluble protein fraction of lens. Protein carbonyl groups of soluble protein fraction of different groups were assayed by reactivity to 2,4-DNPH as described in the Methods section. Data are mean±SD (n=4). Different superscripts above the bars denote that data are significantly different among the groups.

A wide variety of structurally diverse sugar-derived AGE have been demonstrated in cataractous lens and the predominant antigenic AGE are argypyrimidine, carboxyethyllysine, carboxymethyllysine [5-8,39-41]. Having shown the effective reduction of HMW aggregates in the soluble fraction of lens upon feeding ginger, we evaluated the immunoreactivity of some of these AGE using antibodies raised against CML-BSA, MGO-BSA, and AGE-BSA in the soluble as well as insoluble portion of lens protein. While immunoreactivity of glucose derived AGE (AGE-BSA) was found in the soluble portion (Figure 5), the presence of MGO-BSA and CML could be detected in the insoluble protein fraction (Figure 6 and Figure 7). Detection of increased AGE in the insoluble fraction of diabetic lenses indicates that formation of AGE on lens proteins led to aggregation and insolubilization of proteins, finally resulting in cataract formation. Feeding of rats with ginger reduced the formation of AGE in both soluble and insoluble protein fraction suggestive of its antiglycating action (Figure 5, Figure 6, and Figure 7). Ginger was also effective against glyco-oxidative damage to the eye lens as feeding of ginger could decrease protein carbonyls which are increased in diabetic lens soluble protein fraction (Figure 8).

### Polyol pathway

Dicarbonyls such as glyoxal and MGO, produced as an intermediate of the Maillard reaction, are substrates of aldose reductase (ALR2) [42], known to activate the polyol pathway under hyperglycemic conditions. In addition, induction of ALR2 by AGE [43] indicates that accelerated formation of AGE in vivo may elicit the activation of the polyol pathway. Hence, having shown the reduction of AGE, we evaluated the polyol pathway of lens upon ginger feeding. Specific activity of polyol pathway enzymes ALR2 and SDH was significantly increased in untreated diabetic animals compared to control rats. Feeding ginger to diabetic rats resulted in normalization of ALR2 and SDH activities (Table 3). However, we did not find significant inhibition of ALR2 in vitro with aqueous extract of ginger in comparison to other aqueous extracts [44]. Hence, probably the reduction of carbonyl mediated stress (Figure 8) by ginger might be responsible for restoring ALR2 activity after feeding ginger. Further, these findings are in concordance with previous studies demonstrating a cross-talk between these pathways [37,38,41,42].

**Table 3:** Effect of ginger on specific activity of aldose reductase (ALR2) and sorbitol dehydrogenase (SDH) in rat lens.

**Enzyme**	**Group I**	**Group II**	**Group III**	**Group IV**
ALR2	34.12±1.8^a^	53.67±2.3^b^	41.46±3.6^c^	37.1±3.18^a^
SDH	2.40±0.3^a^	3.78±0.6^b^	2.77±0.2^c^	2.44±0.2^a^

Aldose reductase activity was expressed as μmoles NADPH oxidized/h/100 mg protein. SDH activity was μmoles NADH oxidized/h/100 mg protein. The data are mean±SD (n=3). Different superscripts denote that data are significantly different among the groups.

## Discussion

Globally, the number of people with diabetes is projected to rise from 171 million in 2000 to 330 million in 2030 [1,2,45]. It has been estimated that India will have the largest number of diabetic subjects in the world by the year 2025 [1,2,45]. Taking these projections into account, diabetes could become a major threat to public health. Although, there have been major advances in the control of hyperglycemia (diabetes) through dietary changes, hypoglycemic agents, insulin, and islet transplantation, the management of long-term complications of diabetes, such as blindness due to cataract and retinopathy, remain serious problems to be dealt with. The impact of glycemic control in the prevention of diabetic complications has been established by studies like the UK Prospective Diabetes Study and the Diabetes Control and Complications Trial. Nevertheless, perfect glycemic control is not always possible. Further, persistence of progression of hyperglycemia-induced complications during a subsequent period of normal glucose homeostasis (called as memory of glucose toxicity) [46], suggest that exclusive management of glucose can no longer be viewed as sufficient for the control of long-term complications. Hence, agents, which can prevent diabetic complications irrespective of glycemic control, would have advantages in the management of secondary complications.

Several mechanisms have been proposed to explain accelerated cataract formation due to diabetes [47,48]. Glycation induced structural damage to lens proteins resulting in the formation of light scattering aggregates and ALR2 mediated osmotic stress have both been considered as predominant factors. Previously we have identified some natural sources, including ginger, for their potential to inhibit these pathways with the ultimate goal to prevent or treat diabetic complications [23,24,30,32,44]. In this study we demonstrated that the antiglycating effect of ginger could be translated to delay the onset and progression of cataract in experimental diabetic rats.

The rhizome of ginger contains over 20 phenolic compounds. The major active principles include zingiberene, bisabolene, gingerols, and shogaols. These compounds have been reported to display diverse biologic activities such as antioxidant, anti inflammatory, anticarcinogenic, antidiabetic, hypoglycemic, hypolipidimic, and aldose reductase inhibitory properties [25-29,44,49]. Multiple properties of these active components could also be responsible for the observed effect in the present study in addition to its antiglycating potential that we have attributed as a predominant mechanism for the delay of diabetic cataract.

Previous studies reported that active component of ginger inhibited ALR2 and prevented the accumulation of galacitol in the galactose induced cataract [50]. We also found that the aqueous extract of ginger showed both antiglycating activity and ALR2 inhibition [23,44]. Nevertheless, ginger seems to modulate both glycation and polyol pathways because of a cross-talk between these pathways. However, antiglycating potential, not only in vitro but also in vivo, appears to be predominant over ALR2 inhibition [23,44]. Thus, the antiglycating nature of ginger could be the major mechanism in ameliorating STZ-induced diabetic cataract. Our earlier studies with curcumin, cumin and tannoid-enriched fraction of *Emblica* have shown that antioxidant, antiglycating and ALR2 inhibition were partly responsible for the delay of diabetic cataract in rats [24,30,32]. Interestingly, unlike the previous studies, besides delaying the progression and maturation of cataract a significant delay in the onset of cataract by ginger was noticed in this study. This could be attributed to its ability to prevent the multiple changes associated with the accumulation of AGE, i.e., reduction in the carbonyl stress, inhibition of osmotic stress by reducing the activity of polyol pathway, and prevention of oxidative stress [38].

In conclusion, results of the present study suggest that agents or compounds that exert multiple actions like antiglycating, ALR2 inhibition, antioxidant, and antidiabetic/hypoglycemic properties might provide a viable approach, either food based or pharmacological, in the treatment of diabetic complications.
